# Using stable isotope (*δ*^13^C, *δ*^15^N) values from feces and breath to infer shorebird diets

**DOI:** 10.1007/s00442-022-05257-x

**Published:** 2022-09-20

**Authors:** Tomohiro Kuwae, Jun Hosoya, Kazuhiko Ichimi, Kenta Watanabe, Mark C. Drever, Toshifumi Moriya, Robert W. Elner, Keith A. Hobson

**Affiliations:** 1 Coastal and Estuarine Environment Research Group, Port and Airport Research Institute, 3-1-1, Nagase, Yokosuka, 239-0826 Japan; 2 Japanese Bird Banding Association, 115, Konoyama, Abiko, 270-1145 Japan; 3 Seto Inland Sea Regional Research Center, Kagawa University, 4511-15, Kamano, Aji, Takamatsu 761-0130 Japan; 4 Environment and Climate Change Canada, Pacific Wildlife Research Centre, 5421 Robertson Road, Delta, BC V4K 3N2 Canada; 5 Japan Bird Research Association, 1-29-9, Sumiyoshi-Cho, Fuchu, 183-0034 Japan; 6 Department of Biology, University of Western Ontario, London, ON N6A 5B7 Canada; 7 Wildlife Research Division, Environment and Climate Change Canada, Saskatoon, SK S7N 3H5 Canada

**Keywords:** Discrimination factors, Droppings, Blood, Feathers, Tissues

## Abstract

The use of stable isotopes of carbon (*δ*^13^C) and nitrogen (*δ*^15^N) from feces and breath offers potential as non-destructive tools to assess diets and nutrition. How stable isotope values derived from breath and feces compare with those from commonly used tissues, such as blood fractions and liver, remains uncertain, including understanding the metabolic routing of dietary nutrients. Here, we measured *δ*^13^C and *δ*^15^N from feces and *δ*^13^C of breath from captive Red-necked Stints (*Calidris ruficollis*) and 26 species of wild-caught migratory shorebirds (*n* = 259 individuals) and compared them against isotopic values from blood and feathers. For captive birds fed either cereal- or fish-based diets, differences in *δ*^13^C between feces and lipid-free diet were small, − 0.2 ± 0.5‰ and 0.1 ± 0.3‰, respectively, and differences in *δ*^15^N, − 0.7 ± 0.5‰ and − 0.5 ± 0.5‰, respectively. Hence, *δ*^13^C and *δ*^15^N values from feces can serve as proxies for ingested proteinaceous tissues and non-soluble carbohydrates because isotopic discrimination can be considered negligible. Stable isotope values in plasma and feces were strongly correlated in wild-caught shorebirds, indicating feces can be used to infer assimilated macronutrients. Breath *δ*^13^C was 1.6 ± 0.8‰ to 5.6 ± 1.2‰ lower than bulk food sources, and breath C derived from lipids was estimated at 47.5% (cereal) to 96.1% (fish), likely underlining the importance of dietary lipids for metabolism. The findings validate the use of stable isotope values of feces and breath in isotopic assays to better understand the dietary needs of shorebirds.

**Supplementary Information:**

The online version contains supplementary material available at 10.1007/s00442-022-05257-x.

## Introduction

Knowledge of diet is key to understanding species interactions, food web structure, nutrient physiology, and biogeochemical cycling (Martínez del Rio et al. 2009; Estes et al. 2011; Atwood et al. 2015; Hoenig et al. 2021). Dietary needs have been investigated using various conventional approaches, including the identification of dietary items ingested, their digestion and assimilation (Nielsen et al. 2018). The measurement of naturally occurring stable isotopes (*δ*^13^C and *δ*^15^N) of food and consumer tissues has become a widely used proxy for assessing diet (Fry 2006). Accordingly, proteinaceous tissues, including muscle, liver, and blood fraction are commonly collected from animals for *δ*^13^C and *δ*^15^N analyses (e.g., Hobson and Clark 1992a, b). However, catching wild animals is often difficult and harvesting tissue from captured individuals can be intrusive.

The analysis of excreta presents a non-intrusive means of inferring diet because such material often contains a large fraction of undigested material (Des Marais et al. 1980; Podlesak et al. 2005; Salvarina et al. 2013). Generally, sampling excreta is straightforward, although individual- and population-based identification of each excreta is complicated by the problem of associating excreta with known individuals. Excreta do, of course, represent short-term dietary integration. Similarly, isotopic analysis of breath samples is relatively non-invasive compared to other techniques and represents recently metabolized diet, providing an indication of short-term energy pathways or stored macronutrients such as lipids or glycogen (Hwang et al. 2007; Hobson et al. 2009; Salvarina et al. 2013; McCue and Welch 2015). Feces and breath may serve for isotopic analyses in cases where either diet remains constant through time or a time series of material is available. However, to date, feces and breath have not been commonly used for dietary analyses of wild animals, and various aspects of their application require further examination, including the role of metabolic routing (Hobson et al. 2022) and potential isotopic discrimination between ingested diet and the resulting feces produced.

The full implementation of stable isotopic techniques to decipher diets requires knowledge of isotopic values of both dietary inputs as well as the isotopic discrimination, or isotopic difference, between diet and the tissues produced on that diet (*Δ*^13^C and *Δ*^15^N). Also, knowledge of metabolic routing, or the preferential allocation of elements from macronutrients to ultimate tissue synthesis, is crucial but seldom considered (Podlesak et al. 2005; Hatch et al. 2002a; McCue and Welch 2015). The use of stable isotope measurements of tissues to infer diet is well established in birds (Hobson 2011; Karnovsky et al. 2012; Hoenig et al. 2021). However, determining trophic discrimination factors between animal diet and tissues is influenced by several considerations, including individual functional traits, resource availability, and isotope routing (Martínez del Rio et al. 2009), and remains challenging especially when using bulk tissue analyses (Post 2002). While fecal samples have been used for research into the diets of wild birds (Podlesak et al. 2005; Salvarina et al. 2013), they have not been subjected to validation using isotopically controlled diets, and the assumption of previous researchers has been that *Δ*^13^C and *Δ*^15^N between the bulk food sources and the feces, at least for shorebirds, are negligible (Kuwae et al. 2008, 2012). Investigating the relative proportions of ingested but unassimilated dietary items and their macronutrient composition under controlled feeding conditions can determine the extent to which such an assumption is valid (Sponheimer et al. 2003) and how data from wild birds should be interpreted.

A necessary refinement to isotopic methodology for deciphering diets is the consideration of metabolic routing. Here, understanding the various metabolic pathways involved between diet and tissue synthesis is required. Tissue *δ*^15^N analyses only provide information on dietary protein because lipids and carbohydrates contain little nitrogen. In contrast, carbon in tissues can enter by various pathways and be routed from all macronutrients into all tissues (Karasov and Martinez del Rio 2007; Whiteman et al. 2012). Thus, ideally, trophic discrimination factors linking dietary macronutrients with consumer tissues should be known (Podlesak et al. 2005; Ben David et al. 2012; Hobson et al., 2022).

In this study, we investigated isotopic linkages between diet and feces, breath, feathers, and blood under diets differing in isotopic values of macronutrients using captive and wild shorebirds. First, captive Red-necked Stints, *Calidris ruficollis,* housed in a controlled tidal flat experimental mesocosm (Kuwae and Hosokawa 2000; Kuwae 2005) were used for a controlled-diet experiment. These birds were fed either a cereal- or fish-based diet of known bulk isotopic composition, and the isotope values of feces and breath measured. Second, we compared the isotope values of feces, blood, and feathers taken from captive birds held under standardized conditions in the mesocosm for 4 years. Third, associations between blood isotope values and those of feces were measured in a variety of wild shorebird species captured during migration on the east coast of Japan. The overall objective was to assess to what degree isotopic measurements of feces and breath samples could replace isotopic analyses based on blood or other tissues/samples to infer diet in shorebirds. The research allowed the isotopic consequences of metabolic routing of macronutrients, in particular, lipids, from diet to these tissues/samples to be assessed. Improved understanding of the natural variation of isotopic discrimination factors between individual traits such as body mass, irrespective of species, could allow a more general application of our findings to a broad spectrum of avian diet studies.

## Methods

All work was conducted in accordance with applicable regulations in Japan (e.g., Protection and Control of Wild Birds and Mammals and Hunting Management Law, the Act on Welfare and Management of Animals) and all possible efforts were made to minimize stress on the shorebirds.

### Captive shorebirds in a tidal flat mesocosm

#### Mesocosm

**Fig. 1:**
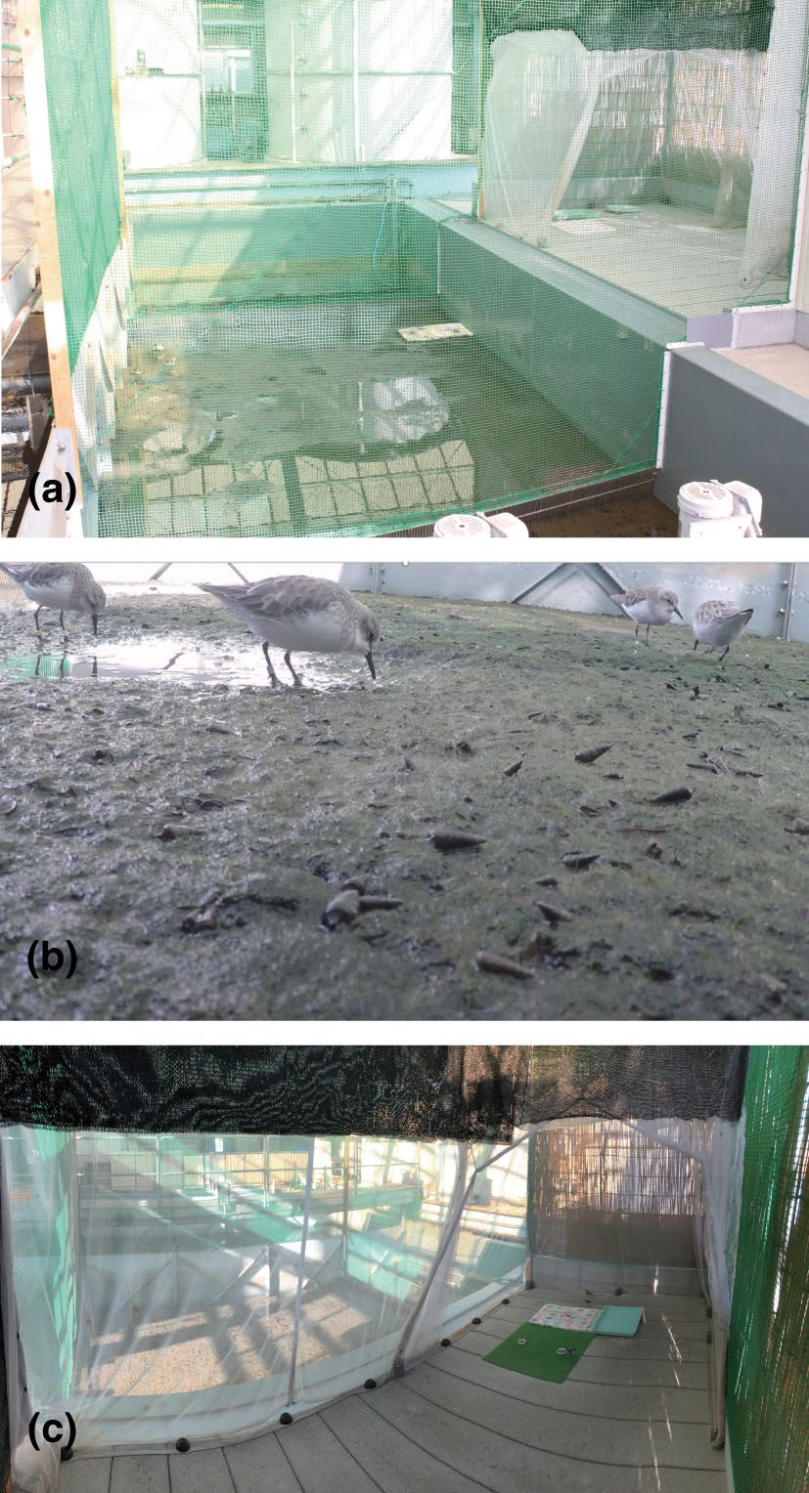
The tidal flat experimental mesocosm at the Port and Airport Research Institute, Japan (also see Supplementary Movie). For detailed specifications of the mesocosm, see Kuwae and Hosokawa (2000). **a** Birds are reared in cages with green netting. **b** Red-necked stints (*Calidris ruficollis*) feeding on invertebrates and biofilm in the experimental ecosystem and a cereal-based pellet. **c** The birds fed only on either cereal-based or fish-based pellets during the diet-controlled experiments in the inner white cage (no invertebrates and biofilm). Pellets are in the white vessel on the greenish artificial turf

First-year juvenile Red-necked Stints were captured using mist nets at the Torinoumi intertidal flat (38°1.8′ N, 140° 54.9′ E), Miyagi, Japan, during their southward migration in September, 2013 (Permit No. 1308061, Japanese Ministry of Environment, August, 6, 2013). Birds were transferred and reared (Registration No. 14B “a” 1910–1922, Kanagawa Prefecture, Japan, September 2, 2013) at the tidal flat mesocosm, Port and Airport Research Institute (Kuwae and Hosokawa 2000; Kuwae 2005) (Fig. 1, see also Supplementary videos). The experiment was started with five acclimatized individuals (Individual bird ID: Birds A–E, see Supplementary Table 1). During the four-year experiment, captive birds fed on naturally occurring biofilm and macroinvertebrates in the mesocosm (Kuwae and Hosokawa 2000; unpublished data), plus a commercial cereal-based pellet diet.

In January 2019, a controlled-diet experiment was performed to examine how breath *δ*^13^C values would vary with changes in macronutrient content of the diet. Providing diets that differed in macronutrient composition (Table 1) enabled us to determine whether diet types affected trophic discrimination factors and metabolic routing (see Supplementary Materials for the macronutrient analyses of diets). Three of the focal birds (A–C) that had been held on the commercial cereal-based diet were isolated in an enclosure (inner cage, Fig. 1) and began receiving a fish-based pellet for a period of one week, during which breath samples were taken from birds on days one, two, and five of the experiment (see Supplementary Data File [Table 1_IFEF_breath]). After the switch period, birds were returned to the cereal-based pellet, and breath samples were taken from birds for four consecutive days. Food and water were available ad libitum throughout the acclimation and experimental periods, and no decrease in body masses occurred during the experiment.

**Table 1:** Macronutrient composition of the pellets used in the diet experiments with captive Red-necked Stints (*Calidris ruficollis*)

	Carbohydrates			Proteins			Lipids		
	% to total C			% to total C			% to total C		
	mean	SD	*n*	mean	SD	*n*	mean	SD	*n*
Cereal-based pellet	45.7	1.5	5	31.0	0.5	5	23.4	1.8	5
Fish-based pellet	27.7	1.9	5	58.2	0.8	5	14.1	2.3	5

#### Fecal sampling and preparation

Approximately 20 droppings excreted by 4–5 individuals (October 2013–May 2016: Birds A–E; September 2016–January 2017: Birds A–D) were collected each sampling day (*n* = 394 droppings), without an individual bird ID being assigned to each dropping (see Supplementary Data File [Table 2_IFEF_feces]). For the controlled-diet experiments, three individuals (Birds A–C) were isolated in an enclosure (inner cage, Fig. 1) and droppings (*n* = 77) collected after they received only a cereal-based pellet for two days, without an individual bird ID for each dropping (see Supplementary Data File [Table 1_IFEF_feces]) or only a commercial fish-based pellet (*n* = 20), without an individual bird ID for each dropping for another two days (see Supplementary Data File [Table 1_IFEF_feces]). For both cases, sampling commenced two days after isolation, allowing the droppings to reflect the particular pellet diet (Kuwae et al. 2008). Only fresh droppings were collected and immediately stored at − 20 °C until drying.

Droppings were pre-treated prior to stable isotope analyses to extract the fecal portion and remove isotopically fractionated metabolites, such as urea and ammonium, as well as carbonates (Kuwae et al. 2008, 2012). Feces (i.e., the fecal portion extracted from a dropping) and the two types of pellets used in the experiments described above were dried at 60 °C for 24 h, then ground to powder with a mortar and pestle. Subsamples of each powdered sample (ca. 5 mg) were placed in micro-tubes, mixed with a 1.4 ml 2:1 chloroform/methanol solution, centrifuged for 15 min at 1300×*g*, and the supernatant eliminated. The treatment was repeated four times. In the process, lipids and soluble carbohydrates (e.g., sugars) were also removed from the samples. The effect of the treatment on the isotope values of the two pellet types was also examined. Next, dropping samples were acidified using 1 M HCl to eliminate carbonates because of the sediment content in feces.

#### Feather sampling and preparation

Feathers were collected for deriving discrimination factors between different tissues/samples taken from individual birds experiencing similar conditions. As the collected feathers had dropped naturally, rather than being plucked, we were not able to match individual feather to the originating bird. Primary feathers (*n* = 4 in November, 2014; *n* = 4 in February, 2015) and breast feathers (*n* = 3 in November, 2014; *n* = 3 in February, 2015) were collected from the enclosure housing five second-year individuals (Birds A–E) during the period when all their juvenile feathers were being replaced by adult feathers (see Supplementary Data File [Table 3_IFEF_feather]). As such, we expected feather stable isotope values to reflect fully the provisioned diet treatments during the 4-years experiment. Feathers were stored at − 20 °C until being treated by soaking in a 2:1 chloroform/methanol solution overnight, decanted and air-dried to remove surface oils. Then the feather samples were powdered with a mortar and pestle prior to analyses.

#### Blood sampling and preparation

Whole blood was taken from five individuals (Birds A–E) in June 2015. Approximately, 150 µL of blood was collected from the brachial vein in the wing with a sterilized 27G S.B. 3/4″ needle (Terumo, Tokyo, Japan) and transferred to a 250 uL heparinized capillary tube (Drummond Scientific Company, Broomall, Pennsylvania, USA, SKY-I KN3131665). The blood was centrifuged for 5 min at 7000 rpm (AcNo Light; Sagami Co., Ltd, Yokohama, Japan) to separate fractions of plasma and red blood cells, before being immediately transported to the laboratory and stored at − 20 °C until drying (see Supplementary Data File [Table 3_IFEF_blood]). Plasma and red blood cell samples were dried at 60 °C for 24 h, then ground to powder with a mortar and pestle prior to analyses.

#### Breath sampling and preparation

**Fig. 2:**
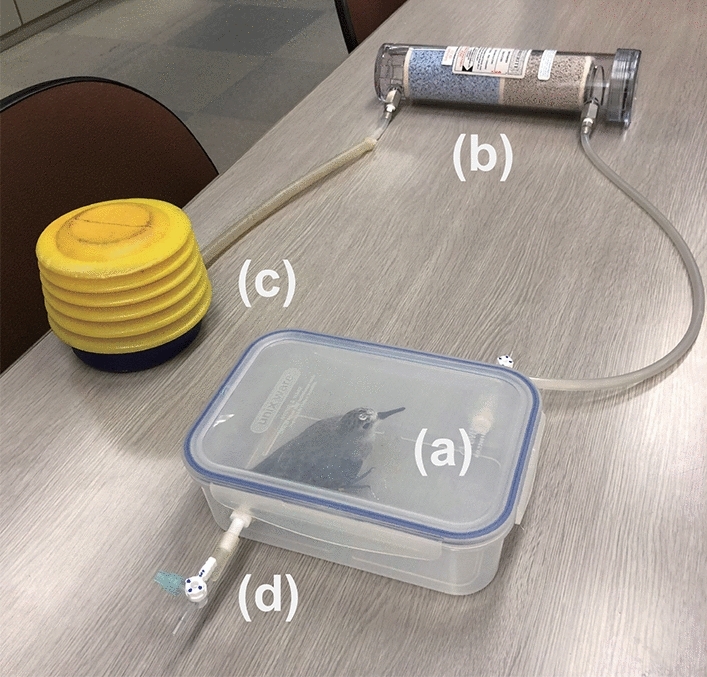
The device for breath sampling. **a** Airtight plastic container, **b** drierite and ascarite filter to generate CO_2_-free air, **c** hand pump to force air through the filter, and **d** gas sampling port

Breath sampling was conducted by placing a bird in an airtight plastic container that could be flushed and filled with CO_2_-free air (using a hand pump to force air through a drierite and ascarite filter) before being isolated via stopcocks for three minutes, allowing bird breath CO_2_ to accumulate (Fig. 2). Next, air from the container was subsampled through a sampling port using a gas-tight syringe and immediately introduced into an isotope-ratio mass spectrometer, described below. We confirmed that there was no contamination of breath samples with ambient CO_2_ by a controlled experiment, in which measurements were taken with no birds in the containers and no CO_2_ was detected.

### Wild bird sampling

Twenty-six species of shorebirds, a total of 259 individuals (Supplementary Table 2), were captured by mist nets on the Torinoumi intertidal flat on their stopover during both northward and southward migrations (August 2012–November 2014) (Permit No. 120124001-42, 1302275, 1401301, 1503262, 1604181, and 1705171, Tohoku Regional Environment Office, Japanese Ministry of Environment). Following capture, each bird was tagged with an individually numbered leg band and aged (juvenile: until the first northward migration, adult: after the second southward migration) based on plumage characteristics and morphometric measurements (Higgins and Davies 1996). We confirmed that there were no recaptures based on the presence or absence of a leg band. Each captured bird was placed in a plastic case lined with a plastic bag and left for < 30 min to allow the collection of individual-based droppings. The fresh droppings were expected to reflect recent diet (minutes to hours, Kuwae 2007; Kuwae et al. 2008). Next, approximately 150 µL of blood was collected from a subsample of the captured birds, and part of the samples were centrifuged to separate fractions of plasma and red blood cells, using the same protocols described above, before the birds were released (see Supplementary Data File [Table 4_Torinoumi]). All the samples were transported on ice for < 2 h and stored at − 20 °C until drying, dried at 60 °C for 24 h, then ground to powder with a mortar and pestle prior to analyses.

### Stable isotope analyses

Samples were weighed into tin foil capsules (ca. 1 mg), crushed and introduced into a zero blank autosampler under He flow. Next, samples were combusted at 1000 °C, and the gases separated in a Thermo Flash elemental analyzer (Thermo Instruments, Bremen, Germany) via a Conflo III device interfaced with a Thermo DELTA Plus Advantage continuous-flow isotope-ratio mass spectrometer at the Port and Airport Research Institute (Nagase, Yokosuka, Japan) to determine *δ*^13^C, *δ*^15^N, and the carbon/nitrogen elemental ratio (C/N ratio). Vienna Pee Dee Belemnite (VPDB) and atmospheric AIR were used as reference materials for *δ*^13^C and *δ*^15^N, respectively. Within-run laboratory calibration standards were L-Histidine [*δ*^13^C -VPDB =  − 10.18‰, *δ*^15^N-Air =  − 7.74‰; Shoko Co., Ltd., Minato-ku, Japan]; L-Alanine [*δ*^13^C -VPDB =  − 19.6‰, *δ*^15^N-Air = 10.1‰; Shoko Science Co., Ltd., Yokohama, Japan]; and Glycine [*δ*^13^C -VPDB =  − 32.3‰, *δ*^15^N-Air = 1.12‰; Shoko Science Co., Ltd.]). The long-term within-run analytical precision of the system was within ± 0.2‰ for both *δ*^13^C and *δ*^15^N.

### Statistical analyses

*Diet-controlled experiments in mesocosm.* We used a General Linear Model (GLM) to examine whether the *δ*^13^C of breath values differed depending on input diets, and whether *δ*^13^C values differed between the diets themselves. The model had the *δ*^13^C value as the response variable, with explanatory variables being the source of the measure, namely, bird breath (cereal diet), cereal (bulk), cereal (lipid removed), bird breath (fish diet), fish (bulk), and fish (lipid removed). We used a GLM to test for average differences in *δ*^13^C values between breath samples under different diets and between diets themselves.

Breath samples collected from the same birds may be considered statistically non-independent replicates, so, to examine the potential for any effects of pseudoreplication, we additionally used a General Linear Mixed-effects Model (GLMM; Bates et al. 2015) to analyze the *δ*^13^C data based on the breath samples alone (without including the samples from the diet items). The GLMM included random effects for individual ID and sample date, effectively controlled for different breath samples being taken from the same birds, analogous to a repeated measures design. The residual SD from the GLMM had a value of 0.93, higher than the bird ID random effect of 0.33, and the date SD value with a 0.82, meaning samples taken from the same bird were more variable than samples taken from different birds or from a different date. This pattern is consistent with the high C turnover in breath (Podlesak et al. 2005). Thus, we retained the GLM approach, with the understanding that some interdependence may exist between some replicates, but it was not sufficient to alter the main conclusions (that birds on different diets have different breath *δ*^13^C values).

*Wild-caught birds*. The *δ*^13^C and *δ*^15^N values of feces and plasma from the wild-caught birds and GLMMs were used to examine whether these *δ*^13^C and *δ*^15^N values varied by age of bird (adult vs juvenile, categorical variable) and ln-transformed body mass. These GLMMs included species as a random effect to account for non-independence in the data that may have occurred, as individuals within the same species can be expected to have similar isotope values, given they would likely be feeding sympatrically and simultaneously. We did not elaborate on the relationship between feces and blood cells/whole blood because the timing of food reflected was different, that is, feces reflect the diet from minutes to hours before sample collection, and plasma reflects the diet integrated from days before; however, whole blood and blood cells reflect the diet integrated from weeks before (Supplementary Table 4). Thus, comparisons between feces and plasma are reasonable given their comparable turnover rates. In addition, we used GLMMs with species as a random effect to examine whether isotopic discrimination factors (i.e., *Δ*^13^C and *Δ*^15^N) between diet and blood and feces varied by age, body mass, and *δ*^13^C and *δ*^15^N values in feces.

Heteroscedasticity and normality of errors of all GLMMs were assessed according to Crawley (2005). A Gaussian error distribution proved the best model structure and was used for the analyses. Selection of candidate models was based on the principle of parsimony (Burnham and Anderson 2002). We first fitted the global model with all explanatory variables, then used Akaike’s Information Criterion (AIC) to select the most parsimonious model (the smallest AIC) by both backward and forward stepwise variable selection. All statistical analyses were performed using R 4.1.1 (R Core Team 2021).

## Results

### Diet-controlled experiments in mesocosm

#### Food isotope values

**Fig. 3:**
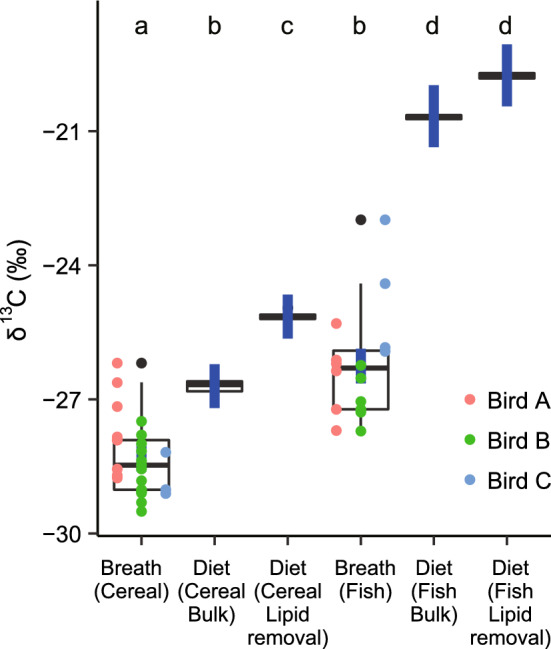
Stable isotope values (*δ*^13^C) of breath samples collected from three captive Red-necked Stints (*Calidris ruficollis*), along with cereal- and fish-based input diets during controlled experiments. Box plot extends from lower quartile to the upper quartile, with median values as a solid bar, and whiskers extend to 1.5 × the interquartile range. Blue points and errors indicate predicted means from a General Linear Model (GLM; see Results). Letters above each box plot denote groups that are statistically different based on least-squared means. Colored data points indicate individual birds in the experiment

As expected, *δ*^13^C values of food items that had undergone lipid removal treatment were significantly higher than those without treatment (Wilcoxon rank sum test, *P* ≤ 0.001, see Table 2, Fig. 3). The C/N ratio with lipids removed was significantly lower than those without lipid removal (Wilcoxon rank sum test, *P* ≤ 0.001). Further, as expected, there was no significant difference between the *δ*^15^N of food with and without lipid removal (*P* = 0.739).

**Table 2:** Stable isotope values (*δ*^13^C and *δ*^15^N) of fecal and breath samples collected from five captive Red-necked Stints (*Calidris ruficollis*) fed either a cereal-based pellet or a fish-based pellet during diet-controlled experiments using a mesocosm

	*δ*^13^C			*δ*^15^N			C/N ratio		
	‰			‰					
	mean	SD	*n*	mean	SD	*n*	mean	SD	*n*
Cereal-based pellets									
Bulk food (a)	− 26.7	0.2	10	6.2	0.1	10	11.4	0.3	10
Food with lipid removal (b)	− 25.1	0.1	10	6.2	0.2	10	9.2	0.2	10
Dietary lipids (c)	− 31.8								
Depletion of lipids to other macronutrients (c–b)	− 6.7								
Feces (d)	− 25.3	0.5	77	5.6	0.6	77	6.9	2.6	77
Feces–food discrimination factor (*Δ*, d–b)	− 0.2	0.5		− 0.6	0.6				
Breath (e)	− 28.3	0.8	25						
Breath–bulk food discrimination factor (*Δ*, e–a)	− 1.6	0.8							
Breath–food lipids discrimination factor (*Δ*, e–c)	3.5								
Fish-based pellets									
Bulk food (f)	− 20.7	0.1	5	10.7	0.2	5	6.2	0.2	5
Food with lipid removal (g)	− 19.7	0.1	5	10.5	0.1	5	5.2	0.3	5
Food lipids (h)	− 26.5								
Depletion of lipids to other macronutrients (c–b)	− 6.8								
Feces (i)	− 19.6	0.3	20	10.0	0.5	20	3.1	0.5	20
Feces–food discrimination factor (*Δ*, g–f)	0.1	0.3		− 0.5	0.5				
Breath (j)	− 26.3	1.2	16						
Breath–bulk food discrimination factor (*Δ*, j–i)	− 5.6	1.2							
Breath–food lipids discrimination factor (*Δ*, j–h)	0.3								

Bulk food values represent the input diets, and discrimination factors (*Δ*) refer to the differences between diets and fecal or breath samples. Dietary lipid *δ*^13^C values were calculated from the bulk diet *δ*^13^C values with lipids and with lipids removed and also had the compositional analyses of the bulk diets (Table 1). Note that multiple breath or fecal samples were taken from each individual bird (see “Methods”). C/N ratio indicates the carbon/nitrogen elemental ratio

#### Feces isotope values

Average body masses of three individuals (Birds A–C) fed cereal-based pellets in September, 2018, ranged from 23.7 to 24.9 g, compared to 27.1 to 28.5 g for the same three birds with fish-based pellets in April, 2017, with 0.6 ± 0.4 g (mean ± SD) as the individual body mass ranges during the experiment.

The *δ*^13^C values of feces closely resembled those of diets with lipids removed (Table 2); the *Δ*^13^C between feces and food with lipid removal was − 0.2 ± 0.5‰ (cereal) and 0.1 ± 0.3‰ (fish), with no significant difference between them (*P* = 0.432 and *P* = 0.083, respectively). Also, the *δ*^15^N values of feces closely resembled those of diets, showing the *Δ*^15^N between feces and food with lipid removal being − 0.6 ± 0.6‰ (cereal) and − 0.5 ± 0.5‰ (fish); however, there was a significant difference between them (*P* = 0.001 and *P* = 0.015, respectively).

#### Breath isotope values

The average body mass of the three individuals (Birds A–C) during the breath experiment ranged from 24.3 to 25.3 g, with 0.5 ± 0.3 g (mean ± SD) the individual body mass range during the experiment, and 0.3 ± 0.2 g (mean ± SD) the difference in mass between the start and the end of the experiments.

The *δ*^13^C values in breath samples differed between birds receiving either a cereal-based or a fish diet (Table 2; Fig. 3). The GLM indicated that *δ*^13^C values differed widely among the six sources (Likelihood ratio test: *χ*^2^ = 40.0, *df* = 6, *P* < 0.001). Breath *δ*^13^C values for birds on both diets were lower than their respective dietary inputs (Fig. 3). The differences (*Δ*^13^C) between breath and bulk food were − 1.6 ± 0.8‰ (cereal) and − 5.6 ± 1.2‰ (fish), and between breath and food with lipid removal was − 3.2 ± 0.8‰ (cereal) and − 6.6 ± 0.5‰ (fish).

We were able to estimate the lipid *δ*^13^C values associated with diet because we measured the bulk diet *δ*^13^C values with lipids and with lipids removed and also had the compositional analyses of the bulk diets (cereal = 23.4% lipid, fish = 14.1% lipid). This resulted in mean estimates for cereal food lipid as − 31.8‰ and − 26.5‰ for the fish diet. Assuming that diet-breath isotopic discrimination during the oxidation of macronutrients in the tricarboxylic acid cycle (TCA) cycle is zero, and that birds were metabolizing only dietary lipids into breath CO_2_, then the corresponding mean *Δ*^13^C for dietary lipid to breath for the cereal diet would be 3.5‰ and 0.3‰ for the mean *Δ*^13^C for dietary lipid to breath in the fish diet. In addition, if birds were metabolizing all the macronutrients, the lipid content of breath is estimated to be 47.5% for the cereal-based diet and 96.1% for the fish-based diet.

### Diet studies in mesocosm

Average body mass of captive birds during the 4-year experiment ranged from 25.9 ± 2.1 g to 28.5 ± 3.2 g (mean ± SD, Supplementary Table 2), with a clear seasonal fluctuation, the highest being around June and the lowest around December (Kuwae et al., unpublished).

The *δ*^13^C, *δ*^15^N, and C/N ratios for feces, blood, and feathers are shown in Table 3. The *Δ*^13^C and the *Δ*^15^N between plasma and feces were − 0.7‰ and 2.7‰, respectively. The *Δ*^13^C and the *Δ*^15^N between blood cell and feces were 1.5‰ and 2.3‰, respectively. The *Δ*^13^C and the *Δ*^15^N between primary feathers and feces were 3.0 ‰ and 3.9 ‰, respectively, and breast feathers and feces were 2.2 ‰ and 3.7 ‰, respectively.

**Table 3:** Stable isotope values (*δ*^13^C and *δ*^15^N) of fecal and breath samples collected from captive Red-necked Stints (*Calidris ruficollis*) feeding on diets of intertidal biofilm and invertebrates, supplemented with cereal-based pellets during diet-non-controlled experiments using a mesocosm

	*δ*^13^C			*δ*^15^N			C/N ratio		
	‰			‰					
	mean	SD	*n*	mean	SD	*n*	mean	SD	*n*
Feces									
All (a)	− 24.3	1.0	394	7.0	0.7	394	5.6	1.8	394
During plasma turnover period (b)*	− 24.5	0.3	18	7.3	0.5	18	6.0	1.6	18
During blood cell turnover period (c)**	− 24.3	0.3	38	7.1	0.5	38	6.1	1.7	38
Blood									
Plasma (d)	− 25.2	0.3	5	10.1	0.2	5	6.2	0.1	5
Plasma–feces discrimination factor (*Δ*, d–b)	− 0.7			2.7					
Blood cell (e)	− 22.8	0.3	5	9.4	0.3	5	3.6	0.1	5
Blood cell–feces discrimination factor (*Δ*, e–c)	1.5			2.3					
Feather									
Primary (f)	− 21.3	0.3	8	10.9	0.3	8	3.5	0.0	8
Breast (g)	− 22.2	0.1	6	10.7	0.3	6	3.6	0.1	6
Primary feather–feces discrimination factor (*Δ*, f–a)	3.0			3.9					
Breast feather–feces discrimination factor (*Δ*, g–a)	2.2			3.7					

Discrimination factors (*Δ*) refer to the differences between feces and breath or blood samples. C/N ratio indicates the carbon/nitrogen elemental ratio. Feather samples may have come from the same birds

^*^Sampled on the same day as the plasma sampling considering the half-life of plasma (up to days)

^**^Sampled over two months prior to the blood cell sampling considering the half-life of blood cells (up to weeks)

### Isotope values in wild-caught shorebirds

**Fig. 4:**
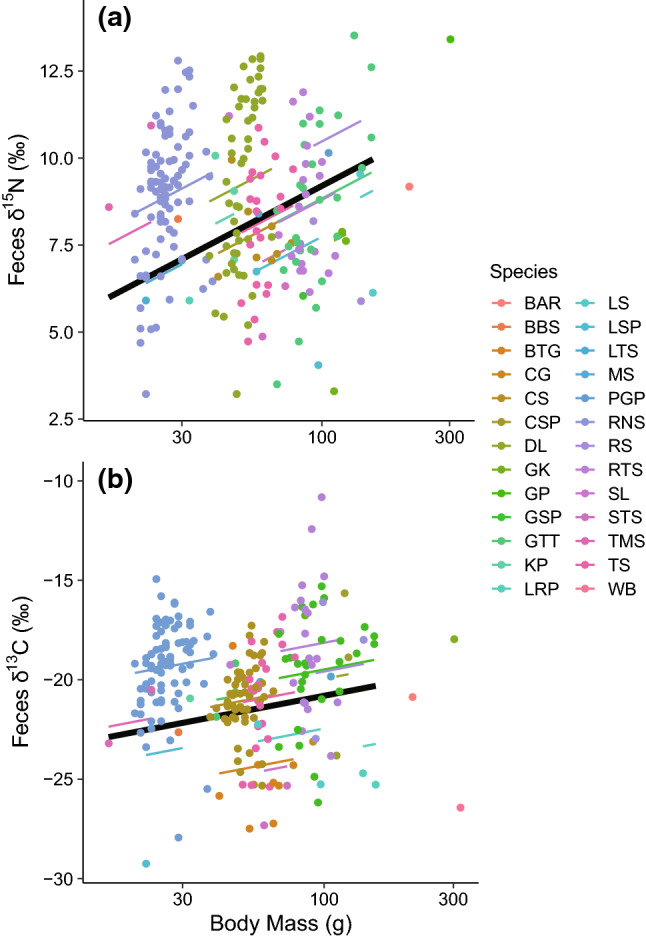
Relationships between *δ*^13^C and *δ*^15^N values (‰) from feces and body weights of individual shorebirds captured during migration at Torinoumi tidal flat, Japan. Solid black lines indicate predicted mean values from a General Linear Mixed-effects Model (GLMM; see Results), and colored lines indicate the species-specific predictions. Species codes are: *BAR* Bar-tailed Godwit, *BBS* Broad-billed Sandpiper, *BTG* Black-tailed Godwit, *CG* Common Greenshank, *CS* Common Sandpiper, *CSP* Common Snipe, *DL* Dunlin, *GK* Great Knot, *GP* Gray Plover, *GSP* Greater Sand Plover, *GTT* Gray-tailed Tattler, *KP* Kentish Plover, *LRP* Little Ringed Plover, *LS* Latham’s Snipe, *LSP* Lesser Sand Plover, *LTS* Long-toed Stint, *MS* Marsh Sandpiper, *PGP* Pacific Golden Plover, *RNS* Red-necked Stint, *RS* Red Shank, *RTS* Ruddy Turnstone, *SL* Sanderling, *STS* Sharp-tailed Sandpiper, *TMS* Temminck’s Stint, *TS* Terek Sandpiper, *WB* Whimbrel. Scientific names are provided in Supplementary Materials Table 1

The average fecal *δ*^13^C and *δ*^15^N values from the 26 species of shorebirds were − 20.2 ± 2.8‰ and 8.7 ± 2.3‰, respectively (Table 4, see Supplementary Fig. 1 for each species value). The *δ*^13^C and *δ*^15^N values of feces were both positively correlated with the body mass of individual birds (Fig. 4). In GLMMs for both isotopes, the residual SD was larger than the SD for the random effect of species (Table 5), indicating that the within-species variance was larger than across species (Supplementary Fig. 1).

**Table 4:** Stable isotope values (*δ*^13^C and *δ*^15^N) of fecal and blood samples collected from wild-caught shorebirds at Torinoumi tidal flat, Japan

	*δ*^13^C				*δ*^15^N			
	‰				‰			
	mean	SD	*n*	*P*	mean	SD	*n*	*P*
Feces (a)	− 20.2	2.8	258		8.7	2.3	259	
Blood								
Plasma (b)	− 19.5	3.0	47		11.7	2.1	48	
Individual-based plasma–feces discrimnation factor (*Δ*, b–a)	0.6	1.6	44	0.021	2.7	1.1	47	< 0.001
Blood cell (c)	− 19.8	3.2	54		9.6	2.1	59	
Individual-based blood cell–feces discrimnation factor (*Δ*, c–a)	0.6	3.1	52	0.254	0.5	1.8	58	0.056
Whole blood (d)	− 17.6	4.3	29		10.6	2.6	29	
Individual-based whole blood–feces discrimnation factor (*Δ*, d–a)	1.5	3.3	28	0.024	1.2	1.7	27	0.001

Discrimination factors (*Δ*) refer to the differences between feces and blood samples

**Table 5:** Statistical results from General Linear Mixed-effects Models (GLMMs) depicting relationships between stable isotope values (*δ*^13^C and *δ*^15^N) of fecal and blood samples collected from wild-caught shorebirds at Torinoumi tidal flat, Japan

Response variable	Variable	Estimate	SE	*P*	*n*
(*R*^2^ value of the best model)		( ±)			
*δ*^13^C of feces (0.48)	Intercept	− 26.02	2.79	< 0.0001	238
	ln(Body mass)	1.13	0.65	0.090	
	Species RE (SD)	2.20			
	Residual (SD)	2.37			
*δ*^15^N of feces (0.37)	Intercept	1.15	2.30	0.620	239
	ln(Body mass)	1.74	0.55	0.003	
	Species RE (SD)	1.39			
	Residual (SD)	2.19			
*Δ*^13^C between plasma and feces (0.29)	Intercept	− 3.45	1.59	0.035	44
	*δ*^13^C of feces	− 0.19	0.08	0.018	
	Species RE (SD)	0.67			
	Residual (SD)	1.39			
*Δ*^13^C between blood cell and feces (0.66)	Intercept	− 13.62	2.71	< 0.001	52
	*δ*^13^C of feces	− 0.64	0.13	< 0.001	
	Species RE (SD)	2.25			
	Residual (SD)	2.04			
*Δ*^13^C between whole blood and feces (0.19)	Intercept	1.54	0.88	0.152	28
	Species RE (SD)	1.47			
	Residual (SD)	3.06			
*Δ*^15^N between plasma and feces (0.63)	Intercept	4.89	0.58	< 0.001	47
	*δ*^15^N of feces	− 0.27	0.06	< 0.001	47
	Species RE (SD)	0.83			
	Residual (SD)	0.79			
*Δ*^15^N between blood cell and feces (0.55)	Intercept	4.27	0.93	< 0.001	58
	*δ*^15^N of feces	− 0.45	0.93	< 0.001	58
	Species RE (SD)	1.18			
	Residual (SD)	1.41			
*Δ*^15^N between whole blood and feces (0.05)	Intercept	1.21	0.37	0.037	27
	Species RE (SD)	0.37			
	Residual (SD)	1.65			

Discrimination factors (*Δ*) refer to the differences between feces and blood samples. Explanatory variables for each model were selected via a bothways stepwise procedure from an initial global model that included ln(Body mass), age (juvenile/adult), and Species as a Random effect (RE). Initial global models for discrimination factors (*Δ*) also included stable isotope values of feces. *n* refers to the number of feces samples used in each model

**Fig. 5:**
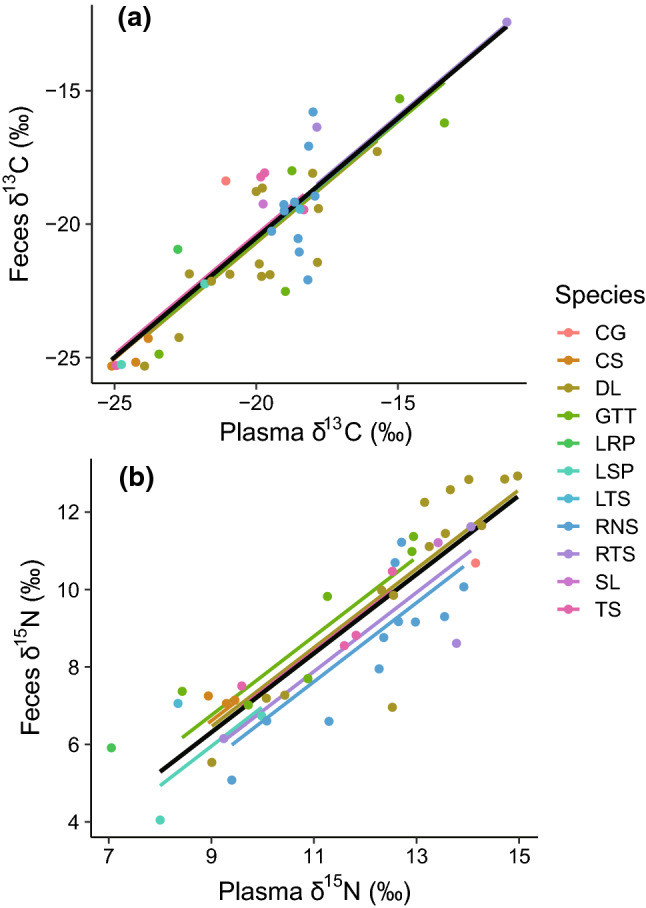
Relationships between *δ*^13^C and *δ*^15^N values (‰) from feces and blood plasma of individual shorebirds captured during migration at Torinoumi tidal flat, Japan. Solid black lines (*δ*^13^C feces =  − 2.599 ± 1.62 + *δ*^13^C plasma × (0.895 ± 0.081)), and **b** between the *δ*^15^N (‰) of feces and plasma of individual birds (*δ*^15^N feces =  − 2.858 ± 0.971 + *δ*^15^N plasma × (1.019 ± 0.083)) indicate predicted mean values from the most parsimonious General Linear Mixed-effects Model (GLMM), and colored lines indicate the species-specific predictions. Species codes: see Fig. 4

Within-individual (paired) *δ*^13^C and *δ*^15^N of feces were significantly different from those of plasma and whole blood (*P* < 0.05) but not significantly different from those of blood cells (*P* = 0.254 and *P* = 0.056, respectively) (Table 4). The within-individual *Δ*^13^C and the *Δ*^15^N between plasma and feces were 0.6 ± 1.6‰ and 2.7 ± 1.1‰, respectively, whereas those between whole blood and feces were 1.5 ± 3.3‰ and 1.2 ± 1.7‰, respectively. Within-individual *δ*^13^C and *δ*^15^N of feces showed strong correlations with those of plasma (Fig. 5).

Isotopic discrimination factors were associated with diets of birds, and did not vary with body mass or age class. GLMM results supported that within-individual *Δ*^13^C and *Δ*^15^N between feces and plasma/blood cells were negatively correlated with *δ*^13^C and *δ*^15^N values of feces (Table 5 and Supplementary Figs. 2 and 3). In contrast, the *Δ*^13^C and the *Δ*^15^N between feces and whole blood were not correlated with *δ*^13^C and *δ*^15^N of feces. Stepwise variable selection in GLMM results did not indicate that any of the *Δ*^13^C and *Δ*^15^N values were associated with body mass or age class of sampled birds.

## Discussion

### Feces samples for diet studies

Our results showed that fecal samples represent a means for reconstructing short-term diets in shorebirds without a need to capture birds. Given both *Δ*^13^C and *Δ*^15^N between feces and lipid-extracted food are small (− 0.6‰ to 0.1‰) in the captive diet-controlled experiment, feces can be used to identify the bulk food sources of ingested proteinaceous tissues and non-soluble carbohydrates for shorebirds (Table 2). Also, feces are advantageous because defecation rates of shorebirds are high, at least during the migratory stopover period when rapid refueling is required (Kuwae et al. 2008; Canham 2020), and thus feces reflect on-site food sources and reducing the carry-over effect from former staging sites (Dietz et al. 2010). Moreover, the high fecal production rate of shorebirds may be largely responsible for the strong correlations between isotope values of bulk diet and feces.

When isotopically fractionated nitrogenous metabolites, lipids, and soluble carbohydrates (sugars) were removed from fecal samples by pre-treatment, the *δ*^13^C and *δ*^15^N of feces in our study primarily reflected dietary proteins and non-soluble carbohydrates, such as celluloses. The smaller *Δ*^13^C and *Δ*^15^N values between feces and food with lipid removal, relative to bulk food, clearly indicate that the *δ*^13^C and *δ*^15^N of the treated feces better represented the *δ*^13^C and *δ*^15^N values of ingested proteins and non-soluble carbohydrates.

Our finding that isotope values of feces were similar to lipid-extracted bulk food sources was also supported from the results of *Δ*^13^C and *Δ*^15^N comparisons between feces and feathers for the captive diet non-controlled experiment, and by the *Δ*^13^C and *Δ*^15^N comparisons between feces and blood in the wild-caught birds and captive birds during the mesocosm trials (Tables 3 and 4). All these trophic discrimination factors fell within previously reported ranges for the *Δ*^13^C and the *Δ*^15^N between the diet and tissues/samples with lipid removal (e.g., Peterson and Fry 1987; Hobson and Clark 1992a, b).

The *δ*^13^C and *δ*^15^N of feces have been commonly assumed to be less useful for dietary analyses as feces per se are an aggregation of materials that were either not digested or assimilated. However, in a shorebird study by Kuwae et al. (2008) of the energy budget paired with stable isotope analyses, ca. 25% of the organic matter in the diet was included in the excreta, with no isotope discrimination between this material and the ingested food. Hence, we infer that the macronutrient composition (proteins, carbohydrates, and lipids) of undigested organic matter excreted as feces is not affected by isotope discrimination during digestion and absorption in the digestive tracts of shorebirds (Fry 2006). Similarly, Salvarina et al. (2013) reported no significant difference in *δ*^13^C and *δ*^15^N values among feces with lipid removal treatment, feces without lipid removal treatment, and diet in captive bats (0.1 ± 0.8‰ for *δ*^13^C and 1.5 ± 1.5‰ for *δ*^15^N). In contrast, differences in *Δ*^13^C and *Δ*^15^N between excreta and food have been reported for songbirds (Podlesak et al. 2005) and rodents (Hwang et al. 2007), although no pre-treatment for excreta was applied in these latter studies. Hence, our pre-treatment that removed isotopically fractionated metabolites, such as urea and ammonium, lipids and soluble carbonates, would support its utility for *δ*^13^C and *δ*^15^N analysis of treated feces as a proxy for analysis of lipid-extracted bulk food sources ingested by other avian taxa.

Previous studies on omnivores have suggested that the use of feces incurs the possible bias that less digestible food sources are overrepresented and more digestible food sources underrepresented (Sponheimer et al. 2003; Kuwae et al. 2008, 2012). The commercial pellets used for the present study were formulated for growing birds and contained more digestible food sources compared to wild diets, suggesting that effects of possible overrepresentation of less digestible parts should have been minimized. Overall, while feces cannot provide insight on long-term assimilated diet, we argue that for shorebirds with rapid ingestion and high fecal production rates, fecal isotopic analyses provide a close approximation to recently assimilated diet.

Establishing appropriate trophic discrimination factors between diet and consumer tissues/samples is critical for dietary reconstructions using stable isotope measurements. However, potentially confounding parameters, including metabolic rate, nutritional quality, tissue type, metabolic routing and degree of fasting, also need to be considered (Hobson et al. 1993; Caut et al. 2008a, b, 2009). For our two experimental diets, we found negligible *Δ*^13^C and *Δ*^15^N values between diet and feces. These results are encouraging as they suggest that *δ*^13^C and *δ*^15^N values of suitably treated feces can be used as a proxy for those values in bulk diet. Regardless, before feces can be used to provide direct evidence of assimilation, consistency of stable isotope values should be confirmed between feces and consumer tissues formed through assimilated diet. The latter could be done by sampling feces and blood, as demonstrated here.

Our data from wild-caught birds showed a positive relationship between the *δ*^13^C and *δ*^15^N values of the feces and shorebird body mass, a pattern that was consistent across species (Fig. 4). Possibly, large-bodied birds are more able than smaller counterparts to feed on larger prey that are generally from higher trophic positions (with corresponding higher *δ*^13^C and *δ*^15^N values; Kuwae et al. 2012). Our result that the within-species isotopic variance, that is individual-level variance, was larger than across species (Supplementary Fig. 1) underscores the need to consider individual functional traits, such as body mass, as well as species-level traits.

The observed negative relationships between within-individual *Δ*^13^C and *Δ*^15^N between feces and plasma/blood cells with *δ*^13^C and *δ*^15^N values of feces needs further study (Table 5 and Supplementary Figs. 2 and 3). However, if we assume that *δ*^13^C and *δ*^15^N values of feces represent those of diet, the same relationship has been found for other consumers (Hobson and Clark 1992b; Caut et al 2008a, b, 2009, 2010). One hypothesis that might explain this negative relationship relates to *Δ*^15^N decreasing as dietary protein quality (i.e., the degree to which the diet meets the amino acid requirements of the consumer) increases, and, therefore, *Δ*^15^N decreases with the trophic position (*δ*^15^N) of consumers (Robbins et al. 2005; Caut et al. 2010; but see Pearson et al. 2003).

A clear positive relationship was found between the within-individual *δ*^13^C and *δ*^15^N of feces and plasma for wild-caught shorebirds, independent of age, body mass, and species (Fig. 5). To examine the robustness of this correlation depending on defecation rate, digestibility efficiency, and degree of omnivory, as suggested, we still have to establish the correlation between within-individual *δ*^13^C and *δ*^15^N of feces and plasma across a variety of habitats. However, overall, given that feces sampling is easy, cost effective and non-intrusive, the *δ*^13^C and *δ*^15^N of feces appear practical for short-term diet inferences.

### Breath samples for diet studies

We found that *Δ*^13^C between breath CO_2_ and bulk diets were significantly negative for both the cereal-based (carbohydrate-rich) pellet (− 3.2 to − 1.6‰) and fish-based (protein-rich) pellet (− 6.6 to − 5.6‰); that is, the *δ*^13^C of breath CO_2_ was lower than the bulk food sources (Fig. 3). However, while this result seems contrary to previous studies (McCue and Welch 2015; Whiteman et al. 2012), evaluating which substrate was likely being used for metabolism is critical (Hobson et al. 2022). Fasting animals that switch to metabolize endogenous lipids usually show declines in the *δ*^13^C of breath CO_2_ because lipids are typically depleted in ^13^C by 2–8‰ compared to other macronutrients (DeNiro and Epstein 1978; Peterson and Fry 1987; Hatch et al. 2002b; McCue and Welch 2015). Indeed, the *δ*^13^C of lipids of the food sources, estimated from the results of the macronutrient composition of the pellets (Table 1) and the *δ*^13^C of the bulk food sources and with lipid removal (Table 2), was − 32.2‰ for the cereal-based pellet and − 26.8‰ for the fish-based pellet, with 6.7‰ and 6.8‰ depletion, respectively, compared to the other macronutrients. However, in our case, endogenous lipids and proteins stored in the body were unlikely to have been oxidized and reflected in the *δ*^13^C of breath CO_2_ given that food and water were available ad libitum and body mass did not decrease during the experimental periods. Thus, dietary lipids were likely to have been preferentially oxidized in our study. The lipid content of breath was estimated to be 47.5% and 96.1% for the cereal- and fish-based diets, respectively. The subsequent estimate of mean *Δ*^13^C between dietary lipids from the fish diet and breath CO_2_ of + 0.3‰ was much closer to the results of previous studies that estimated little discrimination between metabolized substrate and breath CO_2_ (Voigt et al. 2008). The higher mean *Δ*^13^C between dietary lipids from the cereal diet and breath CO_2_ of + 3.5‰ reflected use of other macronutrients in addition to lipids for that dietary group. Why lipids were used more than other macronutrients for energy metabolism may be explained by the experiment not being performed during either the pre-migration or molting period when protein demand is high. Nevertheless, our results underline the need to understand which macromolecular substrate is being metabolized when trying to interpret breath CO_2_ *δ*^13^C values (Hobson et al. 2022).

Our findings suggesting selective metabolism of lipids relative to other macronutrients underscore the importance of considering macronutrient routing vs. food sources per se as well as individual ecophysiology (Hobson et al. 2022). In particular, migratory birds vary considerably in their use of dietary carbohydrates, lipids, and proteins from local food versus endogenous stores at stopover and refueling sites (Hobson et al. 2009; Podelsak et al. 2005; Podelsak and McWilliams 2006). In future research, the additional use of the respiratory quotient (RQ) as a means of confirming which macronutrients are being metabolized to CO_2_ will fundamentally advance these captive studies, as a RQ of 1 indicates carbohydrate metabolism, whereas a RQ of 0.7 indicates pure lipid metabolism when no proteins are involved (Brody 1999). Also, there is need to evaluate isotopic discrimination factors between macronutrients and breath CO_2_ and breath *δ*^13^C during a fasted and fed state using ^13^C labeling experiments to establish appropriate trophic discrimination factors for dietary reconstruction.

In summary, stable isotopes of carbon (*δ*^13^C) and nitrogen (*δ*^15^N) derived from feces and breath samples offer potential as non-destructive tools to assess diets and better understand the nutritional needs of staging shorebirds and, possibly, other animals. The *δ*^13^C and *δ*^15^N values from feces with lipid removal provide a valid proxy for ingested proteinaceous tissues and non-soluble carbohydrates. Similarly, stable isotope values in plasma and feces were strongly correlated, indicating feces in addition to plasma may also be used to infer assimilated macronutrients. These results suggest that the isotopic reconstruction of diets of shorebirds can be greatly simplified and made less invasive.

## Supplementary Information

Below is the link to the electronic supplementary material.

Supplementary file1 (DOCX 398 KB)

Supplementary file2 (XLSX 20 KB)

Supplementary file3 (XLSX 68 KB)

## Data Availability

The datasets generated during the current study are available in the Electronic Supplementary Material.
