# Supplementary material for: Using stable isotope (δ13C, δ15N) values from feces and breath to infer shorebird diets
Source: Oecologia. 2022 Sep 20;200(1-2):23–35. doi: 10.1007/s00442-022-05257-x (PMC9547797; doi:10.1007/s00442-022-05257-x)
Supplement: Supplementary file 1 — Supplementary file1 (DOCX 398 KB) [file 442_2022_5257_MOESM1_ESM.docx]

**Supplementary material**

**Pellet macronutrient analyses**

Carbohydrate, protein, and lipid contents in the pellet were analyzed using the elemental analyzer and the methods described in the main text. To quantify lipids, samples were treated with the chloroform-methanol extraction method. Because this treatment extracts almost all lipids and a part of soluble carbohydrate and proteins, we estimated the extracted amounts by the following equations:

*W*_Lip_ + *W*_Carb_ + *W*_Prot_ = *W*_Extr_ (1)

(*N*_pre_ – 0.173 × *W*_Prot_) / (100 – *W*_Lip_ – *W*_Carb_ – *W*_Prot_) = *N*_post_ /100 (2)

(*C*_pre_ – 0.776 × *W*_Lip_ – 0.529 × *W*_Prot_ – 0.444 × *W*_Carb_) / (100 – *W*_Lip_ – *W*_Carb_ – *W*_Prot_) = *C*_post_ / 100 (3)

where, *W*_Extr_, *W*_Lip_, *W*_Carb_, and *W*_Prot_ are the extracted weight (%) of total amount, lipids, carbohydrates, and proteins. *N*_pre_, *N*_post_, *C*_pre_, and *C*_post_ are the measured nitrogen and carbon contents of pre-treatment and post-treatment samples, respectively. We assumed average nitrogen content of protein (17.3%) and carbon contents of lipids (77.6%), proteins (52.9%), and carbohydrates (44.4%) (Gnaiger and Bitterlich, 1984). Because a unique mathematical solution was not possible using these equations alone, we used the least squares method to estimate the parameters. Total lipid carbon contents were 77.6% of *W*_Lip_ as calculated from this estimation. Total protein carbon contents were quantified from the *N*_pre_ values by using the C/N ratio of 3.6 (blood cell values from this study, see below). Total carbohydrate contents were estimated by subtracting the *C*_pre_ from the sum of total lipids and proteins.

**Other references except for below, see the main text.**

Bearhop, S., Waldron, S., Votier, S. C., & Furness, R. W. (2002). Factors that influence assimilation rates and fractionation of nitrogen and carbon stable isotopes in avian blood and feathers. Physiological and biochemical zoology, 75(5), 451-458.

Evans Ogden, L. J., Hobson, K. A., & Lank, D. B. (2004). Blood isotopic (δ^13^C and δ^15^N) turnover and diet-tissue fractionation factors in captive dunlin (*Calidris alpina* *pacifica*). The Auk, 121(1), 170-177.

Gonzalez, P. M., T. Piersma, and Y. Verkuil. 1996. Food, feeding, and refuelling of Red Knots during northward migration at San Antonio Oeste, Rio Negro, Argentina. Journal of Field Ornithology 67:575–591.

Hatch KA, Pinshow B, Speakman JR (2002) Carbon isotope ratios in exhaled CO_2_ can be used to determine not just present, but also past diets in birds. J Comp Physiol B 172:263–268.

Hobson, K. A., & Clark, R. G. (1992a). Assessing avian diets using stable isotopes I: turnover of ^13^C in tissues. The Condor, 94(1), 181-188.

Hobson KA, Bairlein F (2003) Isotopic fractionation and turnover in captive Garden Warblers (*Sylvia borin*): implications for delineating dietary and migratory associations in wild passerines. Canadian Journal of Zoology 81:1630-1635.

Kuwae, T. (2007) Diurnal and nocturnal feeding rate in Kentish plovers *Charadrius alexandrines* on an intertidal flat as recorded by telescopic video systems. Marine Biology, 151, pp.663-673.

Kuwae T, Beninger PG, Decottignies P, Mathot KJ, Lund DR, Elner RW (2008). Biofilm grazing in a higher vertebrate: the Western Sandpiper, *Calidris mauri*. Ecology 89: 599-606.

Lourenço, P. M., Granadeiro, J. P., Guilherme, J. L., & Catry, T. (2015). Turnover rates of stable isotopes in avian blood and toenails: Implications for dietary and migration studies. Journal of Experimental Marine Biology and Ecology, 472, 89-96.

McCue, M.D. and K.C. Welch Jr. (2015) 13C-breath testing in animals: theory, applications, and future directions. Journal of Comparative Physiology B. DOI 10.1007/s00360-015-0950-4.

Peterson, B. J., & Fry, B. (1987). Stable isotopes in ecosystem studies. Annual review of ecology and systematics, 18(1), 293-320.

Podlesak, D. W., McWilliams, S. R., & Hatch, K. A. (2005). Stable isotopes in breath, blood, feces and feathers can indicate intra-individual changes in the diet of migratory songbirds. Oecologia, 142(4), 501-510.

Salvarina, I., Yohannes, E., Siemers, B. M., & Koselj, K. (2013). Advantages of using fecal samples for stable isotope analysis in bats: evidence from a triple isotopic experiment. Rapid Communications in Mass Spectrometry, 27(17), 1945-1953.

Zharikov, Y., and G. A. Skilleter. 2002. Sex-specific intertidal habitat use in subtropically wintering Bar-tailed Godwits. Canadian Journal of Zoology 80:1918–1929.

**
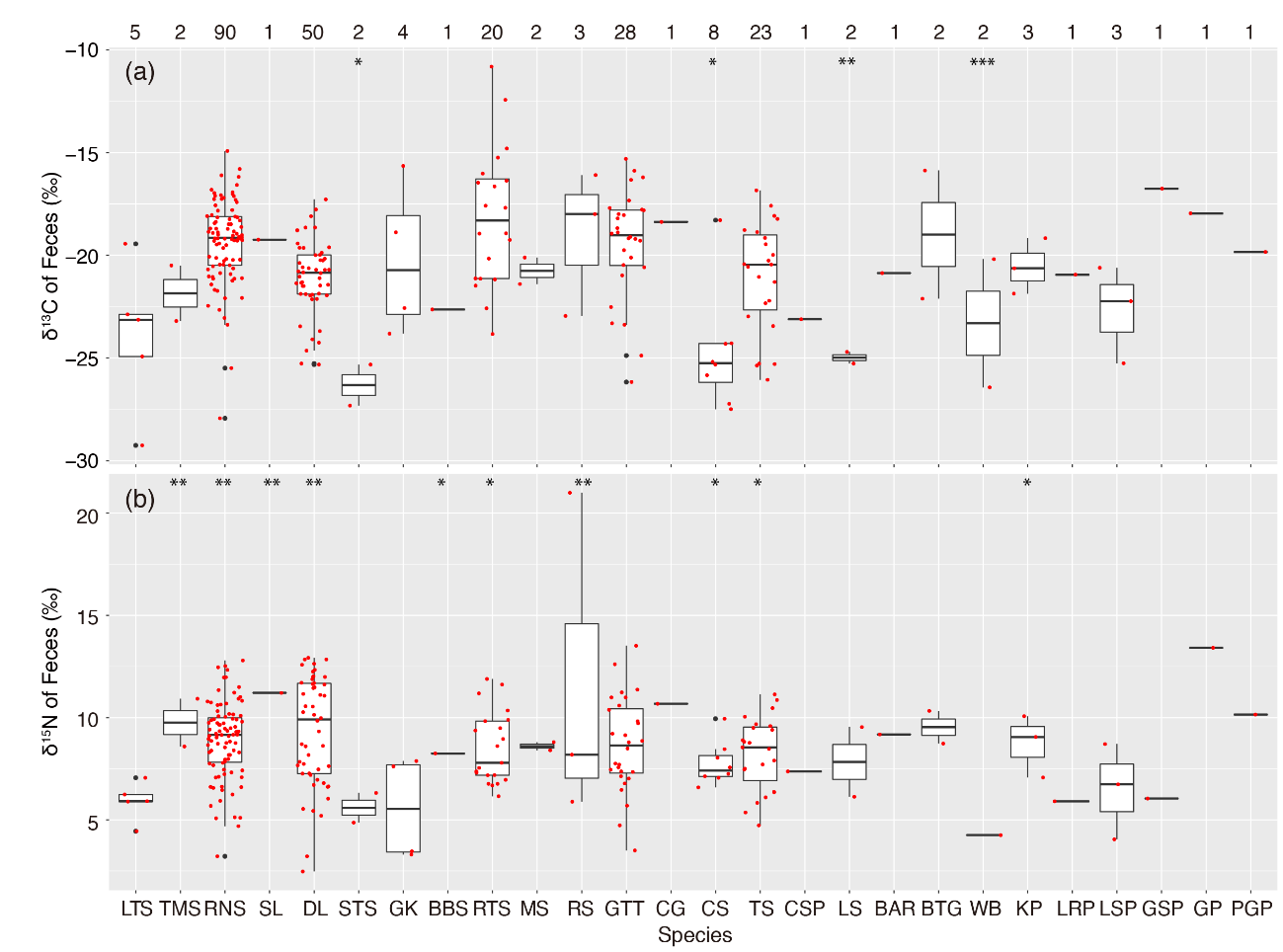
**

**Supplementary Fig. 1** Box plots of the δ^13^C (‰) (a) and δ^15^N (‰) (b) of the feces from Torinoumi tidal flat, Japan. Asterisks indicate the significant difference supported from the GLM model (see **Table 5**) with the level *: <0.05, **: <0.01, ***: <0.0001. Abbreviation of species, see **Supplementary Table 2**. Sample sizes (*n*) are shown at the top.


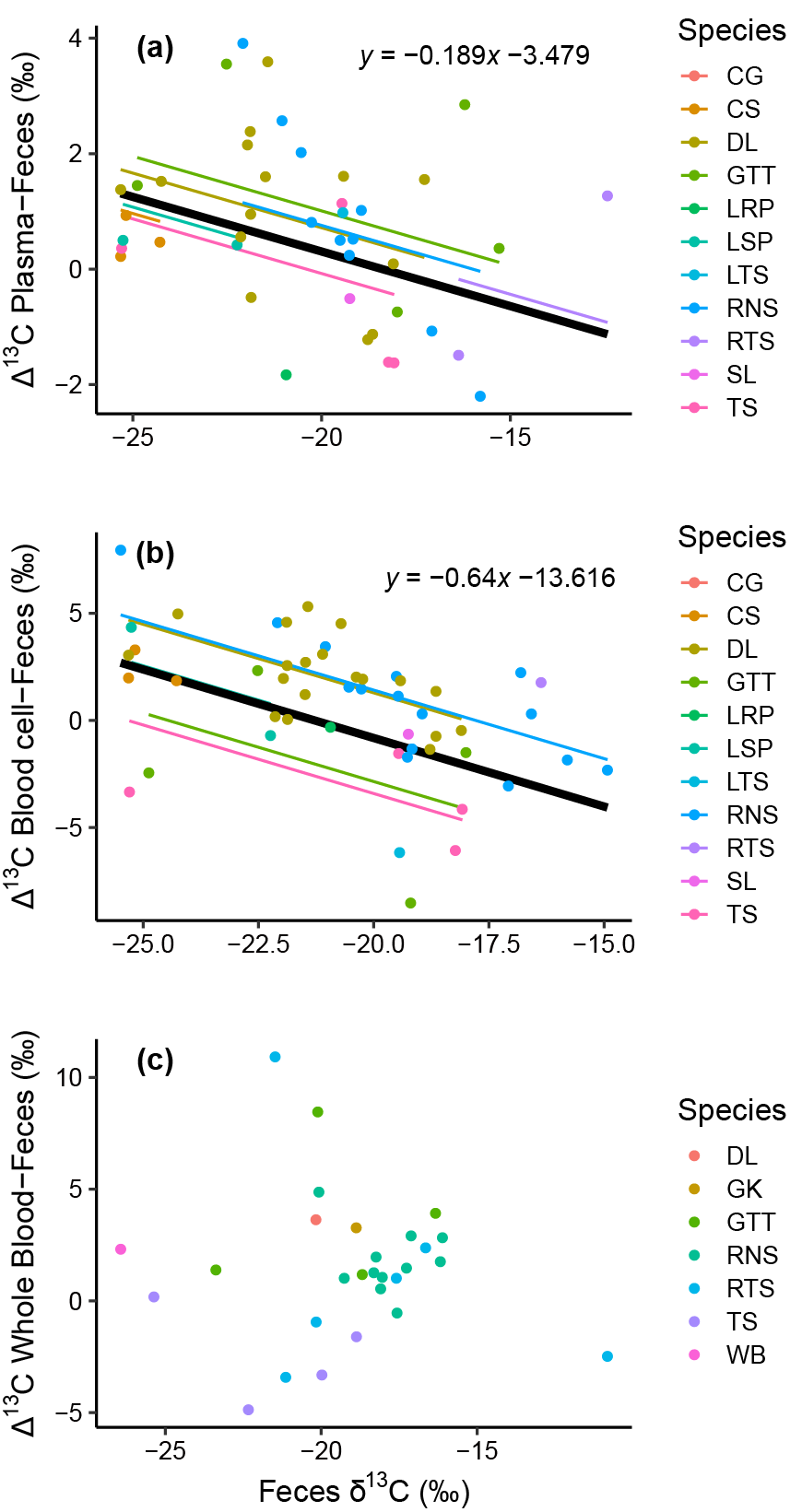


**Supplementary Fig. 2** Statistical results from General Linear Mixed-effects Models (GLMMs) depicting relationships between discrimination factors (Δ) of stable isotope values (δ^13^C) of fecal and blood samples collected from wild-caught shorebirds at Torinoumi tidal flat, Japan. Discrimination factors (Δ) refer to the differences between feces and blood samples. Solid black lines indicate predicted mean values from a General Linear Mixed-effects Model (GLMM; see Results), and coloured lines indicate the species-specific predictions. Species codes as **Supplementary Table 2**.


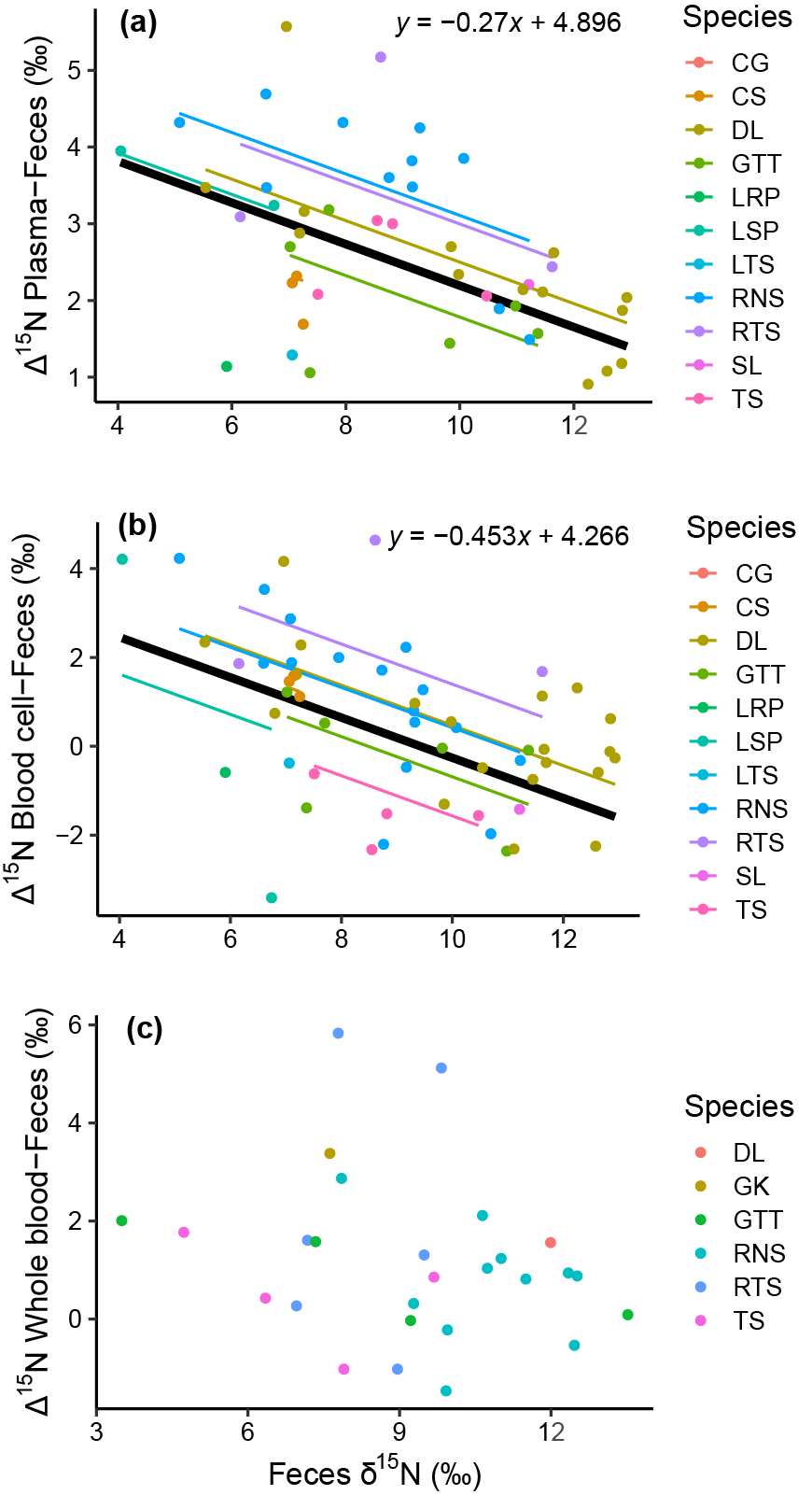


**Supplementary Fig. 3** Statistical results from General Linear Mixed-effects Models (GLMMs) depicting relationships between discrimination factors (Δ) of stable isotope values (δ^15^N) of fecal and blood samples collected from wild-caught shorebirds at Torinoumi tidal flat, Japan. Discrimination factors (Δ) refer to the differences between feces and blood samples. Solid black lines indicate predicted mean values from a General Linear Mixed-effects Model (GLMM; see Results), and coloured lines indicate the species-specific predictions. Species codes as **Supplementary Table 2**.


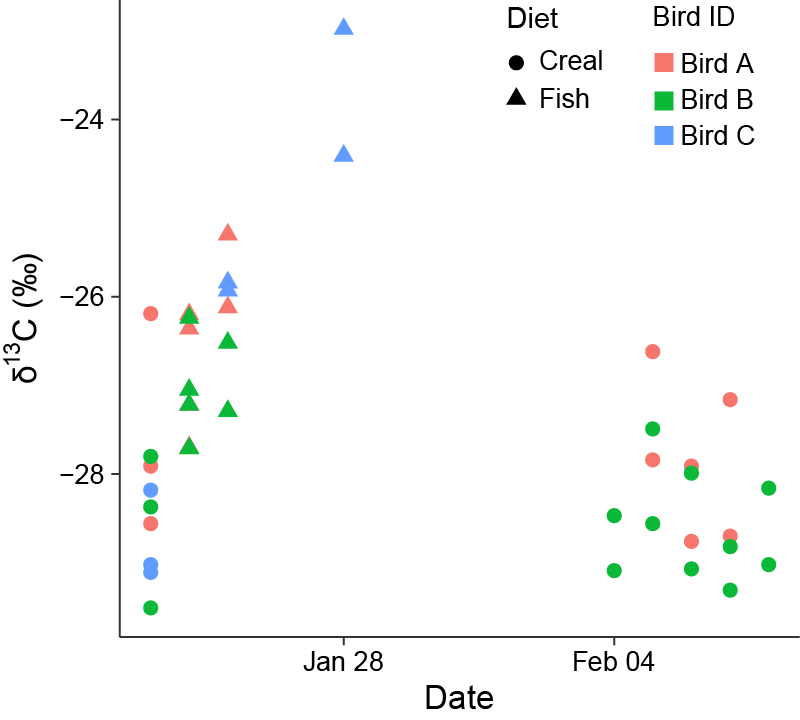


**Supplementary Fig. 4.** Stable isotope values (δ^13^C) of breath samples collected from captive Red-necked Stints (*Calidris ruficollis*) during a diet switching experiment.

**Supplementary Video 1 (IMG_7742.MOV), Movie 2 (IMG_7744.MOV), and Movie 3 (IMG_7745.MOV)**

https://drive.google.com/drive/folders/1K2EGsIuhmfsjnIMzBFL7mSaYc2pu7DO6?usp=sharing

Overview of the tidal flat experimental ecosystem (mesocosm) of the Port and Airport Research Institute, Japan.
